# Quantum size effect on charges and phonons ultrafast dynamics in atomically controlled nanolayers of topological insulators Bi_2_Te_3_

**DOI:** 10.1038/s41598-017-12920-4

**Published:** 2017-10-23

**Authors:** M. Weis, B. Wilk, G. Vaudel, K. Balin, R. Rapacz, A. Bulou, B. Arnaud, J. Szade, P. Ruello

**Affiliations:** 1 A. Chelkowski Institute of Physics and Silesian Center for Education and Interdisciplinary Research,75 Pulku Piechoty 1A University of Silesia, 41–500 Chorzów, Poland; 2 Institut des Molécules et Matériaux du Mans, UMR CNRS 6283, Université du Maine, 72085 Le Mans, France

## Abstract

Heralded as one of the key elements for next generation spintronics devices, topological insulators (TIs) are now step by step envisioned as nanodevices like charge-to-spin current conversion or as Dirac fermions based nanometer Schottky diode for example. However, reduced to few nanometers, TIs layers exhibit a profound modification of the electronic structure and the consequence of this quantum size effect on the fundamental carriers and phonons ultrafast dynamics has been poorly investigated so far. Here, thanks to a complete study of a set of high quality molecular beam epitaxy grown nanolayers, we report the existence of a critical thickness of around ~6 nm, below which a spectacular reduction of the carrier relaxation time by a factor of ten is found in comparison to bulk Bi_2_ Te_3_ In addition, we also evidence an A1g optical phonon mode softening together with the appearance of a thickness dependence of the photoinduced coherent acoustic phonons signals. This drastic evolution of the carriers and phonons dynamics might be due an important electron-phonon coupling evolution due to the quantum confinement. These properties have to be taken into account for future TIs-based spintronic devices.

## Introduction

**Figure 1:**
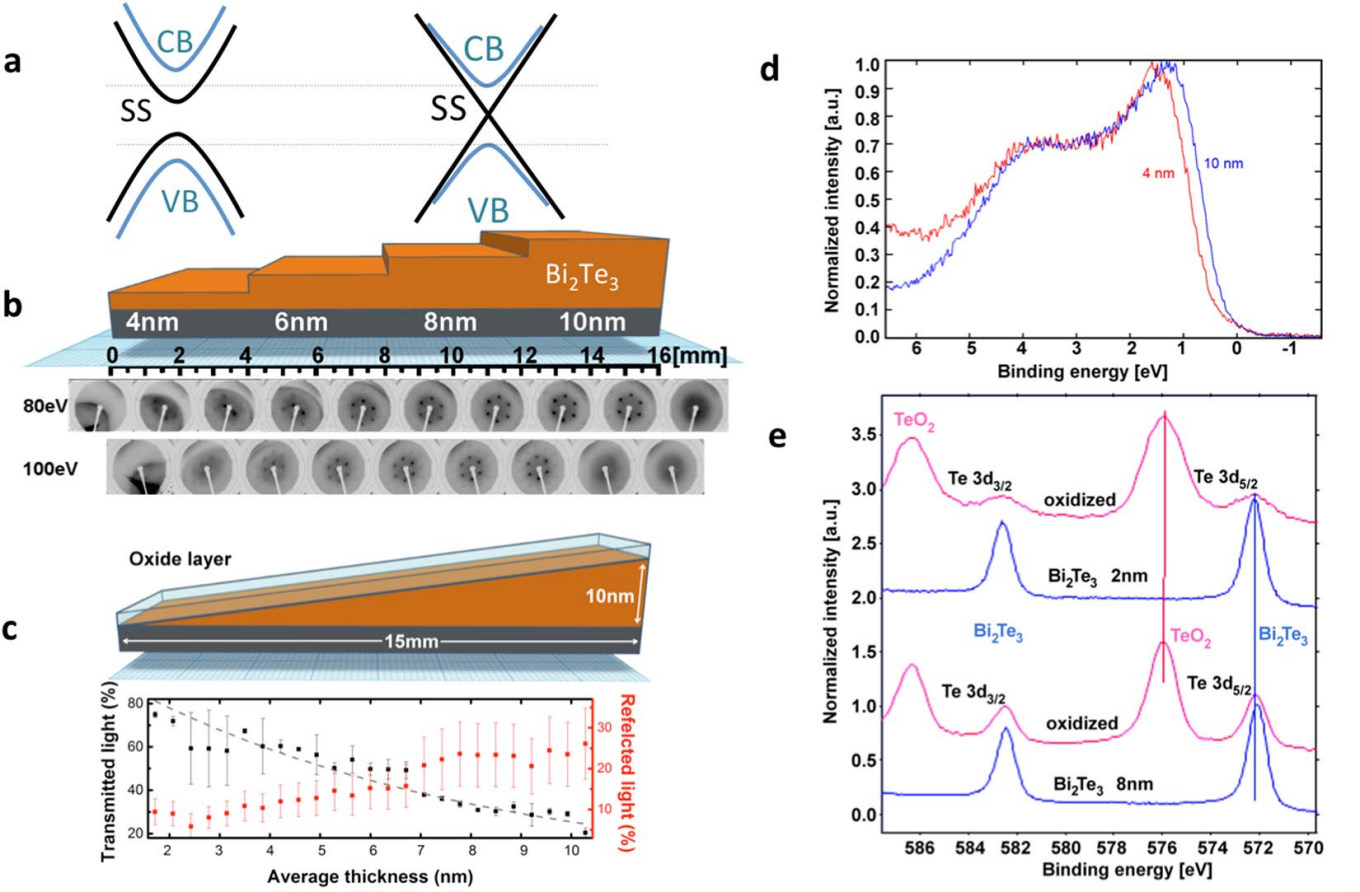
Electronic structure characterization of ultrathin Bi_2_Te_3_ layers with thickness gradient. (**a**) Sketch of the thickness dependence of the bulk and surface electronic states in Bi_2_Te_3_^12^. CB, VB and SS mean conduction and valence bands and surface states respectively. (**b**) Art view of the cross section of the step sample 1 with as inset in the bottom of the figure the thickness dependence of the LEED image obtained for two energies. These images confirm the six-fold symmetry of the z-grown BT layers. (**c**) Art view of the cross-section of the wedge sample together with the continuous wavelength optical transmission (dashed curve is the calculated optical transmission following the Beer-Lambert law, see Methods) and reflectivity along the thickness gradient. (**d**) Valence X-ray photoemission spectra of BT films for variable thicknesses obtained *in-situ* (vacuum) for step sample 2. (**e**) Examples of XPS spectra of the step sample 1 for two thicknesses (8 and 2 nm) obtained for as-grown BT layer (blue curves) and after passivation with oxide cap layer (red curves).

Topological Insulators (TIs) provide new perspectives for next generation spintronics devices thanks to a natural spin polarized surface current that is topologically protected^1,2^. After an intense effort to develop a comprehensive understanding of the bulk properties^1,2^, the age of the investigation of integration of TIs at nanoscale is heralded by now^3^. Among exciting perspectives for nano-spintronics, TIs can be used for charge-to-spin current conversion^3^ or Dirac fermions based nanometer Schottky diode^4^. However, as known in quantum wells, dots or nanocrystals^5,6^ or in metallic nanostructures^7–9^, the downscaling usually leads to apparition of size dependent properties and an enhancement of the quantum confinement effect. In the case of TIs films, it has been reported that the electronic properties are deeply affected indeed under a reduction of the layer thickness where the electronic structure deviates from the bulk one with the lost of surface Dirac states properties^10,11^. A gap opening in the surface Dirac states appears as well as a profound modification of the bulk electronic levels (conduction-CB and valence-VB bands). This phenomenon revealed by Angle-Resolved Photoemission Spectroscopy (ARPES), appears around below 4 nm in Bi_2_Se_3_ (BS) compound^10,11^ and 3–4 nm in Bi_2_Te_3_^12^. A sketch of the thickness dependence electronic structure is shown in Fig. 1a ^12^. Different mechanisms have been discussed to elucidate this phenomenon, including substrate-mediated Rashba effect^10^ and the existence of a crossover from a 3D to a 2D topological insulator^13^. The band gap opening has been reproduced theoretically and it has been reported that additional quantum size effects should appear leading to topological quantum phase transitions that depend on the film thickness^14–16^. While this drastic evolution of the electronic structure submitted to a strong confinement has been well described in TIs at the thermodynamic equilibrium with photoemission spectroscopy^10–12^ and with transport properties measurements^17,18^, the consequence of this confinement on the ultrafast dynamics of carriers and phonons has been debated only recently in BS^19,20^. The ultrafast dynamics of carriers and phonons are only well characterized in bulk crystals of TIs with either time-resolved ARPES or optical methods^21–26^. Moreover, while a theoretical prediction of the enhancement of the coupling between the surface electrons and the acoustic phonons has been reported for a confined structure^27^, no direct experimental reports confirm these predictions so far while ultrafast optics can provide new insights.

In this article, we study the confinement effect on the electronic structure as well as on the ultrafast dynamics of carriers and phonons. We first confirm the appearance of the evolution of the electronic structure for very thin layers with X-ray Photoemission Spectroscopy (XPS) in accordance with what was observed in BS^10,11^ and BT^12^ compounds. Secondly, thanks to femtosecond optical pump-probe methods, we reveal several anomalies on the carriers and coherent phonons ultrafast dynamics in Bi_2_Te_3_ (BT) when the layers thickness is typically smaller than the critical value of ~6 nm. We report indeed a dramatic modification of the hot carriers relaxation time with a reduction from around 2.2 ps (bulk) down to around 200 fs for layer as thin as 4 nm. This transition appears somehow universal since it has been observed for two different samples exhibiting both thickness gradient but having been grown according either to a step or wedge geometry. In parallel to the electron-phonon relaxation time determination, we also report a modification in the spectrum of the photoinduced phonons in the confined nanostructures. While for so-called thick BT layers, the optical phonon mode A1g is clearly generated and detected as expected from bulk behavior, a clear softening and a decrease of its lifetime are observed for ultrathin layers. Surprisingly, while the A1g phonon signal vanishes for ultrathin layers, an increasing contribution of coherent acoustic phonons signal appears with a clear resonant effect. These carriers and phonons dynamics size dependence suggests the existence of a transition in the mechanism of the electron-phonon coupling when reducing the TIs layer which has to be taken into account in the perspectives of the development of TIs nanodevices.

## Results

### Electronic structure of ultrathin BT layers

**Figure 2:**
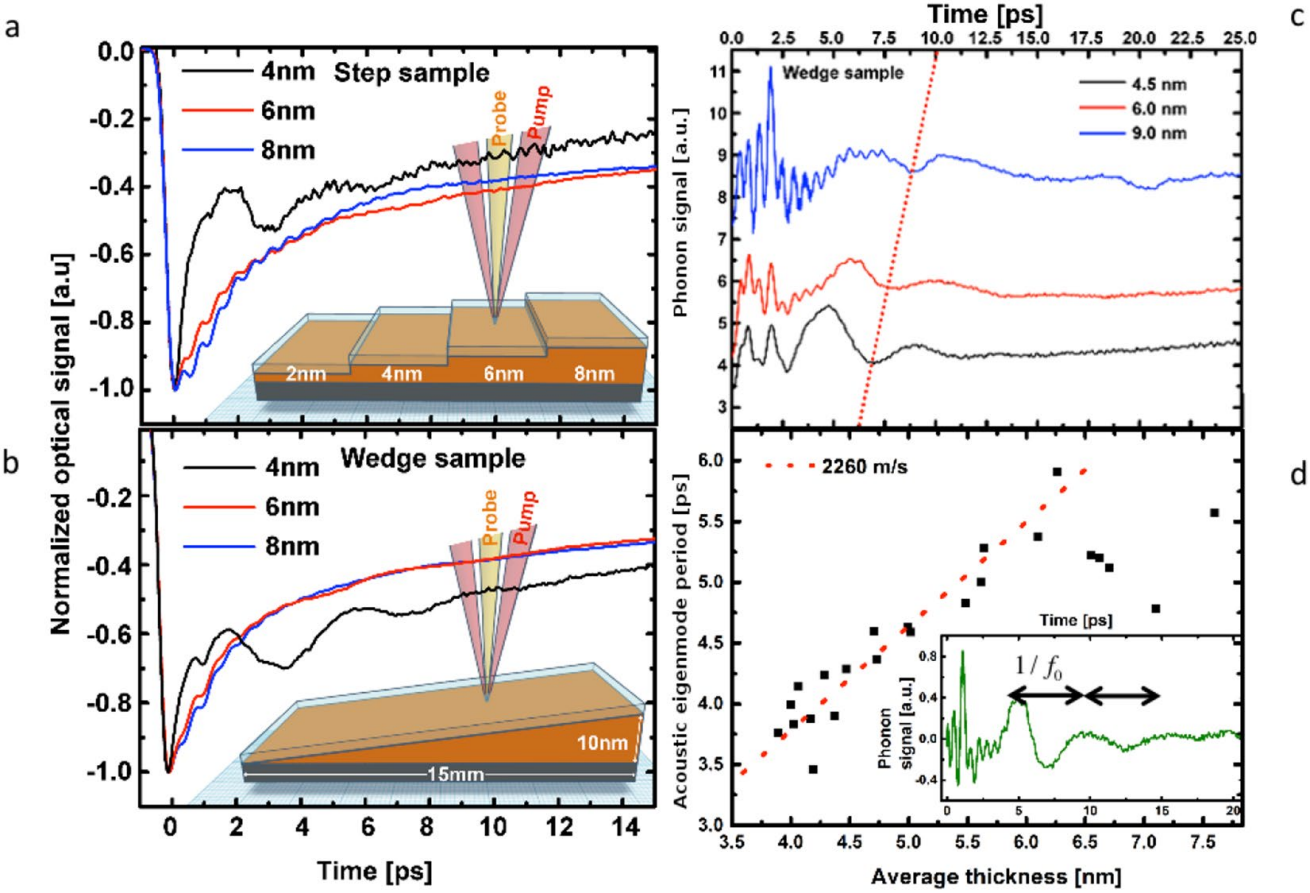
Ultrafast optical response of ultrathin layers of Bi _2_Te_3_.Time-resolved optical reflectivity obtained for various layers of BT for the step (**a**) or the wedge (**b**) sample. The signals are normalized to the maximum of electronic peak. (insets) The pump and probe are represented in the artist view as red and orange beams. (**c**) Contributions of the phonons signals to the transient reflectivity signals for three different thickness. (**d**) Thickness dependence of the longitudinal acoustic resonance eigenmode period $\mathrm{1/}{f}_{{\rm{0}}}$. The slop of the red dashed line provides the estimates of the longitudinal sound velocity (2260 m/s) of BT. Inset shows an example of the typical longitudinal acoustic signal with the period $\mathrm{1/}{f}_{{\rm{0}}}$ for a BT layer thickness of 6 nm.

The investigations were carried out with single crystalline Bi_2_Te_3_ (BT) ultrathin films having thickness varying from around 2 nm up to 10 nm and grown with molecular beam epitaxy with PREVAC system^28^. Different samples were grown with a step or a wedge geometry as shown in Fig. 1b,c (see Methods). Low Energy Electron Diffraction (LEED) image was realized for the step sample (step sample 1) as a function of the BT layer thickness (Fig. 1b confirms the films grow along the c-axis having a six-fold symmetry). Additional LEED analysis are shown in the Supplementary Figures 1–2 of Supplementary Note 1. A wedge sample was also grown and a continuous wavelength optical transmission experiment (bottom of Fig. 1c) was carried out to confirm the thickness gradient of the wedge sample (see Methods). *In-situ* XPS measurements (Fig. 1d) performed on step sample 2 within two regions of 4 and 10 nm of thickness confirms that the electronic structure evolves for ultrathin layer in agreement with the literature^10–12^. The general structure of the valence band is in agreement with the previous experimental results and calculations of total DOS (density of states) for the thick layer (10 nm)^25^. However, a clear difference is visible at the valence band offset where the lowering of the top of valence band by about 0.2 eV with respect to the thicker films is detected for the 4 nm thick film. At the same time the intensity in the vicinity of the Fermi level is unchanged. This is related with the position of the Fermi level which is crossing the bottom of the bulk conduction band. This indicates a n-type doping for our BT films. Regarding the core-levels XPS data (realized on step sample 1 and shown in Fig. 1e), the analysis of the Bi and Te most prominent photoemission lines show one chemical state characteristic for Bi_2_Te_3_^28^ (more complete description of XPS analysis is given in the SI). In Fig. 1e we show the Te 3d_5/2_ line for the as grown film and after the oxidation in air (Bi lines are shown in the Supplementary Figures 3–5 in the Supplementary Note 1). The native and optically transparent oxides layer is formed by TeO_2_ and Bi_2_O_3_ which stabilizes the surface. An angle dependent XPS investigation taking into account the variation of the various components intensity and electron inelastic mean free path have shown that the oxides are on the surface and have a typical thickness of ~2 nm (see SI)^29^. It is important to underline that under surface passivation by the oxides, the core levels (Bi and Te lines) characteristic to BT TIs remain unchanged confirming the stability of the bulk properties of the layer over several months. We are aware that the passivation may affect the surface states^30,31^ since they are very sensitive to a small amount of extrinsic surface defects and are submitted to a rapid aging (few minutes) even in high vacuum. However, we did not observe any changes in the *in-situ* obtained XPS spectra within several minutes and hours after deposition. Consistently with our XPS analysis, we will see in Figs 1 and 2, the ultrafast light-excitation of the A1g Raman active mode of BT films confirming the right structure is obtained. Moreover, the analysis of the evolution in time of the light-excited acoustic phonons (Supplementary Figure 1 in Supplementary Note 1) permits to confirm that the oxide layer is stable in time in agreement with XPS analysis.

### Ultrafast carriers and phonons dynamics

**Figure 3:**
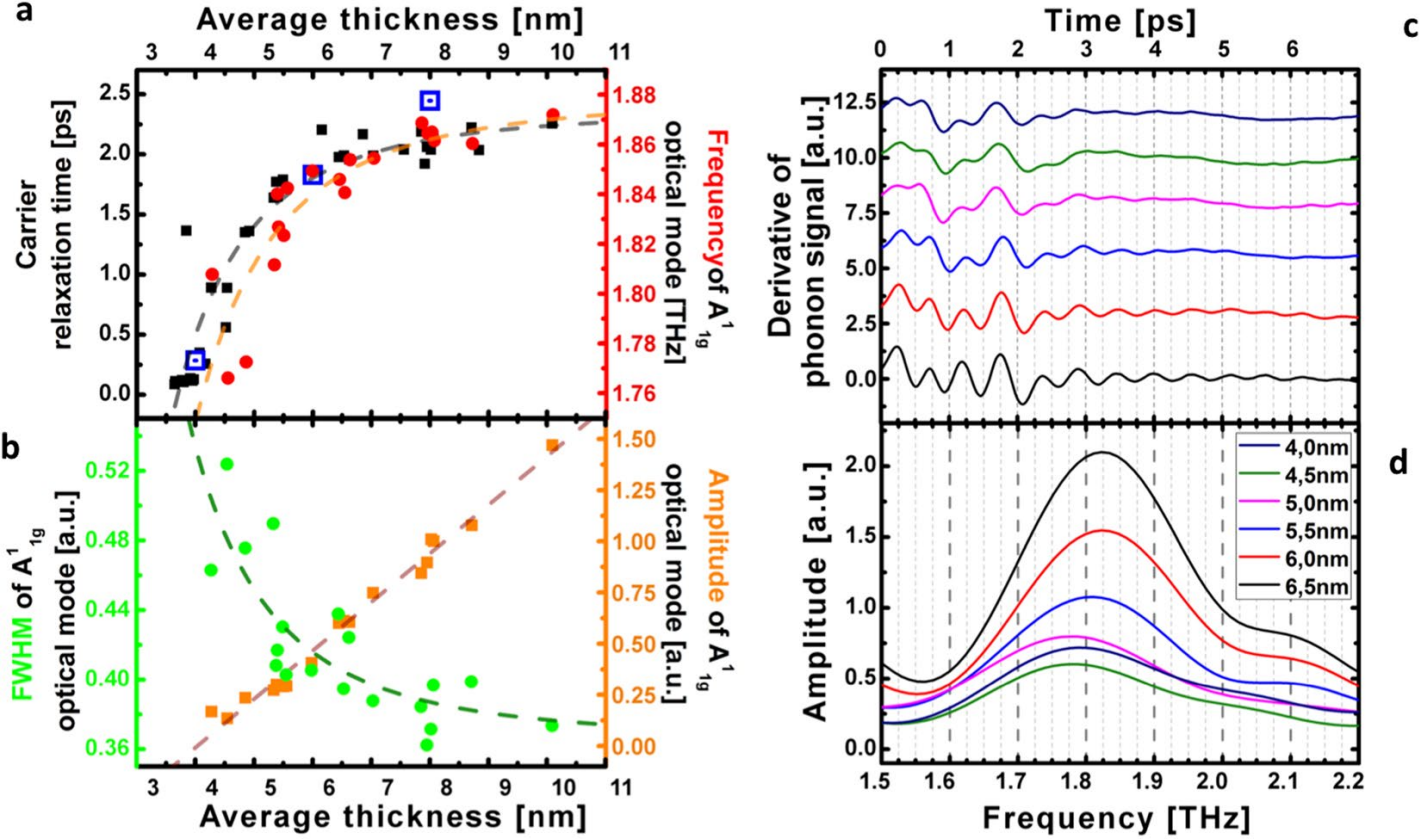
Thickness dependence of the ultrafast carrier and phonon dynamics. (**a**) Thickness dependence of the electron-phonon relaxation time for the wedge (black squares) and step (centred blue squares) samples. Thickness dependence of the A1g optical phonon frequency (red dots). (**b**) Thickness dependence of the A1g optical phonon amplitude (gold squares) and FWHM (green dots) (**c**) Typical oscillatory component at short time scale revealing the A1g optical phonon. (**d**) Fast Fourier Transform (FFT) of signal shown in (**c**) revealing the A1g mode softening for ultrathin layer.

**Figure 4:**
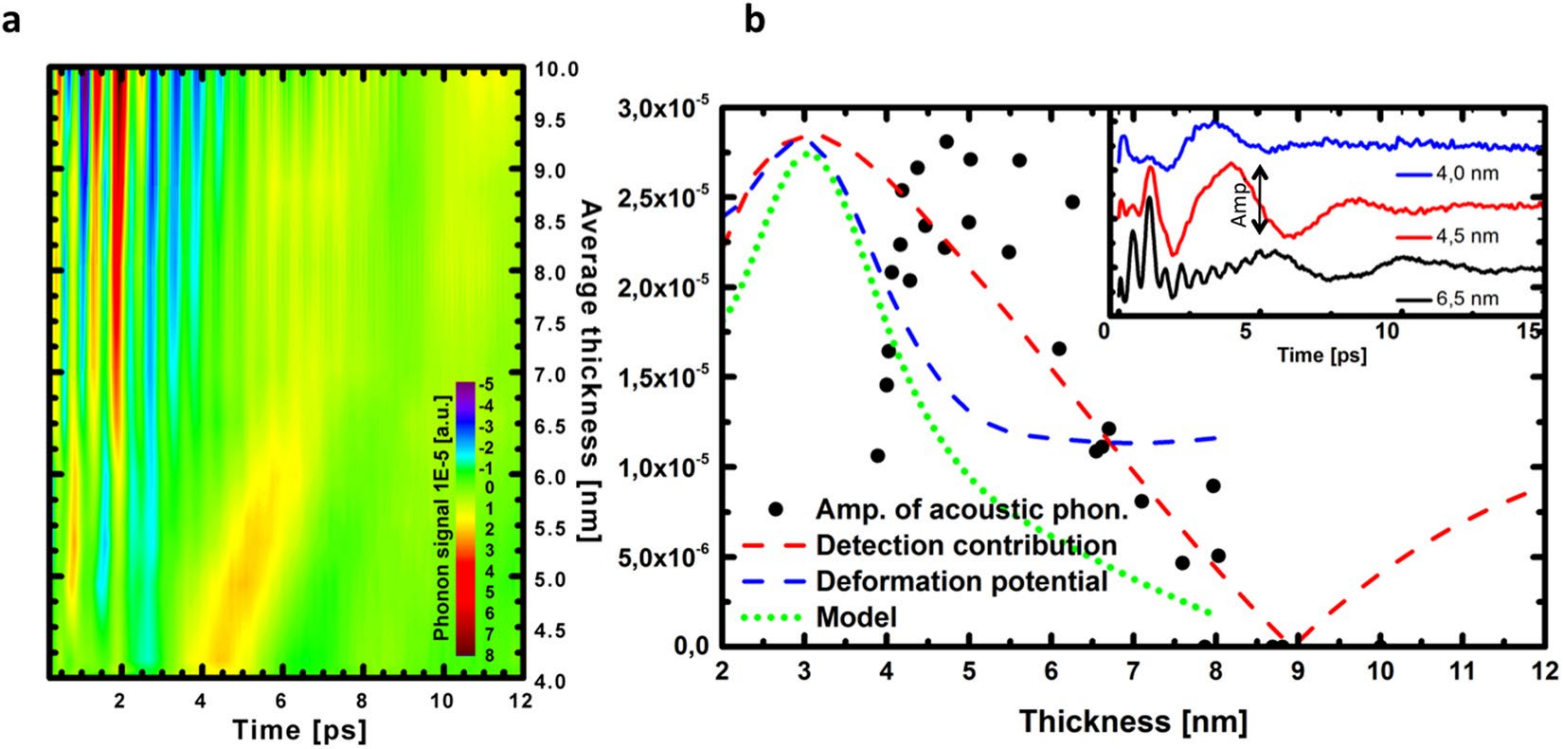
Coherent acoustic phonon generation and detection. (**a**) Map of the optical and acoustic phonon mode amplitudes (fixed pump power) versus time and along the thickness gradient revealing the appearance of larger acoustic phonon signals for ultrathin layers. (**b**) Thickness dependence of the amplitude of the photodetected coherent acoustic signal. A model (green curve) taking into account the detection process and an estimated size dependent electron-hole acoustic deformation potential from the literature^10^ provides a semi-quantitative explanation of the experimental observations.

Some typical time-resolved and thickness dependent optical reflectivity signals are given in Figs. 1a,b for the step sample 1 and the wedge sample respectively. The signals are composed first of a sharp variation of the transient optical reflectivity consecutively to the electronic excitation. Then the decay of the signals is evidenced with, as superimposed, some oscillatory components due to the photoexcitation of phonons (Fig. 1c) that we will discuss latter on. The electronic and the phonons contributions to the transient optical reflectivity were separated as ${\rm{\Delta }}R/R$ = ${\rm{\Delta }}R/R$)_*electron*_ + ${\rm{\Delta }}R/R$)_*phonons*_, where the electronic contribution is composed of a fast rising signal due to the carriers excitation induced by the pump beam followed by a decay described by two exponential functions with a fast decay time ranging from 200 fs to 2 ps (depending on the layer thickness) and a slower component with a time ranging from 30 to 60 ps associated to long living carriers and thermal relaxation process (see Supplementary Note 2 and Supplementary Figure 7 for a detailed example of the fitting procedure). Whatever the step or wedge sample, the first striking evidence we report, is a huge modification of the carrier relaxation time and phonons dynamics (Figs 1–4) for topological insulator films having a very small thickness (typically <6 nm). Moreover, we observed similar behavior when performing the optical transmission measurements (${\rm{\Delta }}T/T$) as shown in the Supplementary Figure 8 indicating the response is really intrinsic to the BT layer. For thick BT layers, the characteristic time of around 2.2 ps is similar to the relaxation of hot carriers (electron and hole) probed in the visible range for bulk Bi_2_Se_3_^21,22^ and bulk Bi_2_Te_3_^26^, while for most confined BT layers, the carrier relaxation time drastically diminishes down to around 200 fs (Fig. 2a). This indicates that the scattering process is close to ten times stronger in the confined BT. We also notice that we were not able to get a clear signal for the thinnest BT layer (2 nm) of the step sample 1. The oscillatory components obtained for thick BT films reveal clearly the A1g mode at 1.86 THz in accordance with the literature^33–36^ (Fig. 2c). An additional modulation of the signal, well visible for the 4.5 nm layer in Fig. 2c (time range 0–2.5 ps), which does not depend on the sample thickness is assigned to the vibration of the top oxide layer (2 nm). While scrutinizing the time-domain optical phonon signals, we evidence a slight optical phonon A1g softening (Fig. 2a,c,d) as well as a decrease of the lifetime (Fig. 2b), confirming that a modification of the interatomic potentials might occur for most confined layers. This softening (~4%) and damping enhancement is consistent with the observations made in Raman scattering measurements where the incoherent A1g phonon exhibits such softening in Bi_2_Se_3_^20,37,38^ and in Bi_2_Te_3_^39^. The fact that this A1g softening occurs both with phonons that are photoexcited with a laser pulse or with thermally excited phonons (Raman spectroscopy) indicates that the softening does not come from hot electrons-driven potential softening as known for bismuth crystal^40,41^. A detailed description of our pump power dependence of the A1g mode is shown in Supplementary Figure 9. As a last observation, our experimental results evidence the appearance of a larger coherent acoustic phonons signal when the layer becomes thin enough (Figs 1c and 2a,b). The spectral analysis of this acoustic phonon signal reveals that the main oscillatory part we observed comes form the excitation/detection of the first acoustic eigenmode of the thin film with ${f}_{{\rm{0}}}=V\mathrm{/2}L$, where $V$ is the sound velocity of the BT layer. The analysis of the eigenmode frequency scales indeed with the inverse of the thickness (Fig. 1d) without any clear anomaly on the sound velocity we estimate at around 2260 m.s ^−1^. However a resonant-like effect is observed in the thickness dependence of the coherent acoustic phonons amplitude as depicted in Fig. 4. The coherent acoustic phonon amplitude (first eigenmode amplitude) clearly increases from 10 to 5 nm while below 5 nm a new regime appears. It is worth to mention that this critical value of 5 nm corresponds quite well to the characteristic thickness below which the carrier relaxation time suddenly falls down (Fig. 2a). As a summary, this set of physical parameters measurements provide a new insight on the ultrafast dynamics of carriers and phonons while submitting the BT compound to a strong confinement. In the next section we discuss the possible fundamental physical origins of these phenomena never reported in TIs BT up to now.

## Discussion

### Carrier relaxation dynamics

The thickness (*L*) dependence of the electronic levels (${\rm{\Delta }}E$) is known in quantum wells and is expected to follow the ${\rm{\Delta }}E=A/{L}^{{\rm{2}}}$ ^42^. This leads in general to size dependent carrier relaxation time with different power of law due to complex dynamics^6^. Reduction of the relaxation time has been reported several time in the literature like in the case of CdSe or CdS nanocrystals^5^ where it is reduced by a factor of 3 (from 270 f s to 85 fs) when the nanocrystal diameter is reduced from 4 nm to 2 nm. Similar decrease of the carrier lifetime ${\tau }_{e-ph}$ was also observed when reducing the diameter of metallic nanoparticles to few nanometers^7,8^. In our case, a numerical adjustment leads to the phenomenological thickness dependent relaxation time as:

1 $${\tau }_{{\rm{e}}-{\rm{ph}}}={\tau }_{{\rm{e}}-{\rm{ph}}}^{{\rm{0}}}-B/{L}^{\alpha }$$

with α = 2.6 and ${\tau }_{{\rm{e}}}^{{\rm{0}}}=2.2$ ps (see dashed red curves in Fig. 2(a) and Supplementary Figure 7). Similar power of law applies well for describing the evolution of the A1g optical mode frequency (${\nu }_{{\rm{A1g}}}$) and its width ${\rm{\Delta }}{\nu }_{{\rm{A1g}}}$ as if a common physical mechanism correlates these parameters. It is important to remind that TIs nanolayers are confined only in one direction while nanocrystals are 3D quantum-confined systems. We know that the electron-hole relaxation time depends both on the matrix element (deformation potential term in the Hamiltonian^43^) and on the final density of states according to the Golden Fermi rule, but the quantitative relationship between this experimental law and the size dependent electronic structure is not an easy task since a complete electronic and phonon dispersion curves are required for bulk and surface states. No complete theoretical formulation of the size dependence relaxation time of the photoexcited population in CdSe and CdS was reported so far due to the same complexity^5^. Different possible contributions can be proposed. When the size of the system is reduced, the carriers surface trapping is known to be more efficient since the probability per unit of time for an electron to reach the surface increase as soon as the mean free path of electrons is comparable with the size of the object. This has been already discussed in the case of semiconductor nanostructures^5^. This surface recombination was one channel of relaxation in semiconductor nanocrystals but quantum size effect on the electron-phonon coupling was also evoked^5^. In the case of metallic nanoparticles, the proposed interpretation was based on the reduction of electron-ion screening interaction due to a decrease of the carrier density close to the surface which contains a non-negligible number of atoms compared to volume ones for few nm nanoparticles radius R^7,8^. A phenomenological scattering rate equation was proposed with $\mathrm{1/}{\tau }_{{\rm{e}}-{\rm{ph}}}=\mathrm{1/}{\tau }_{{\rm{0}}}+{V}_{{\rm{F}}}/\alpha R$, where ${\tau }_{{\rm{0}}}$, *α* and ${V}_{{\rm{F}}}$ are the bulk e-ph relaxation time, an adjustable coefficient and the Fermi velocity^7^. In the case of BS thin films, a decrease of the relaxation time driven by a size dependent Fröhlich interaction was proposed^19^ but many other mechanisms can contribute. In the case of BT nanolayers, we can of course think about possible trapping at the oxide cap layer/BT layer interface which could increase the hot carrier damping, even if it is difficult at that stage to evaluate this contribution. Furthermore, it has been also shown that the range of the bulk-surface interband scattering channel has been estimated to occur up to 5 nm in BT compounds^24^, which matches well the critical thickness we estimate and could indicate that the anomaly we observe is a crossover from the 3D to the 2D system. This is supported by the fact that it is known that the coupling between surfaces Dirac fermions wavefunctions of two opposite sides of a thin layer has been reported to occur over few nm for BS, and even detectable in film of 10 nm of thickness^18^. Moreover, additional effect could be possible such as band bending. It was previously observed at the free surface of a bulk crystal^4^ which means that such situation can be enhanced in ultrathin layers of BT with the closeness of the two opposite surfaces. As a consequence, the hybridization of two opposite surfaces states, the surface band bending, the modification of the bulk electronic levels (see Fig. 1d) as well as the A1g optical phonon softening that we report, are all physical parameters that obviously must correspond to a modification of the electron-ion interaction, i.e the deformation potential parameter, which might influence the carrier relaxation time. The question is why there is an enhancement of the relaxation under the confinement? Beside the surface trapping effect, our observation of an important enhancement of the coherent acoustic phonon signals (Fig. 4a) could be a signature of an enhancement of the electron-hole acoustic phonon deformation potential coupling, which could also contribute to the hot carrier damping rate in the case of electron/acoustic phonon collision processes. This electron-hole acoustic phonon deformation potential is also the driving parameter for the photoexcitation process of coherent acoustic phonon^33^; as a matter of fact, discussing the thickness dependence of the coherent acoustic phonon signal may provide new insight on this electron-phonon coupling strength as presented in the following.

### Electron-hole acoustic phonon deformation potential

The electron-hole acoustic phonon deformation potential is expected to be thickness dependence in topological insulators ultrathin films since the energy bands evolve quite a lot as we observed (Fig. 1c) and accordingly to the literature refs^10,12^). The question is how much this deformation potential varies as a function of the thickness. If we consider that the hot electrons (holes) rapidly thermalize down (up) to the conduction (valence) bands, the relevant deformation parameter is the one close to the band gap (*E*_g_) with:^44–47^

2 $${d}_{33}^{{\rm{ac}}-{\rm{eh}}}=\frac{\partial {E}_{{\rm{g}}}}{\partial {\eta }_{33}}$$

This approximation is justified as soon as the frequency of the detected coherent acoustic phonons is smaller than the inverse of the time of thermalization in the conduction and valence bands respectively with^44,46,47^. As mentioned above in the description of our results, the coherent acoustic phonons under discussion are those corresponding to the thin film eigenmodes, i.e. those inducing an out-of-plane strain ${\eta }_{{\rm{33}}}$. The forth and back bouncing coherent acoustic phonons modulate the entire thickness *L* so that the relevant strain associated to the deformation potential is ${\eta }_{{\rm{33}}}$ =*d* *L/L* /. Consequently the relevant deformation potential becomes:

3 $${d}_{33}^{{\rm{ac}}-{\rm{eh}}}=L\frac{\partial {E}_{{\rm{g}}}}{\partial L}$$

We have access to the thickness dependence of the band gap in the literature with different precisions for BS and BT compounds^10,12^. As a first element of discussion, we have estimated the experimental parameter $L\frac{\partial {E}_{{\rm{g}}}}{\partial L}$ in BS compound based on experimental data^10^ (we used so-called CB1 and VB1 values of ref.^10^. to evaluate the band gap *E*_g_). The thickness dependence is shown (blue dashed curve) in Fig. 3b where we clearly see the enhancement of the deformation potential parameter when reducing the thickness as expected. For the purpose of the comparison we normalize this parameter to the maximum of the experimental amplitude. We are aware that BS and BT are not identical, but this approach shows that an enhancement of the photo induced coherent acoustic phonon could explain partially the experimental observation. There is however a clear deviation for ultrathin layer where the calculated deformation potential cannot explain the sudden decrease of the experimental phonon amplitude. We believe that this effect comes from a detection process that can be numerically estimated as described below. We have realized a calculation of the contribution of coherent acoustic phonons to the transient optical reflectivity (detection process) by considering that none of the physical parameters change but only the thickness is reduced. To do that we have calculated the coherent acoustic phonon contribution to the transient optical reflectivity following the standard method^48–50^. We have performed this calculation by considering the first acoustic eigenmode (${f}_{{\rm{0}}}$) strain field contribution whose standard expression is ${\eta }_{{\rm{33}}}(z,t)=A\times sin[\frac{\pi z}{L}]{e}^{i{\omega }_{{\rm{0}}}t}$ where *A* is the amplitude and ${\omega }_{{\rm{0}}}=2\pi {f}_{{\rm{0}}}$. We can show (see details in Supplementary Note 2) that this contribution to the transient optical reflectivity is given by^48–50^:

4 $$\begin{array}{rcl}{(\frac{{\rm{\Delta }}R}{2R})}_{ac-ph} & \approx  & Re[-4i\times C\times {r}_{12}{k}_{1}\frac{L}{\pi }{e}^{i{\omega }_{{\rm{0}}}t}\\  &  & -\,iC\frac{\partial {k}_{1}}{\partial {\eta }_{{\rm{33}}}}{\int }_{0}^{L}A\times sin[\frac{\pi z}{L}]{e}^{i{\omega }_{{\rm{0}}}t}[{r}_{12}{e}^{-i{k}_{1}(L-z)}\\  &  & +{e}^{i{k}_{1}(L-z)}{]}^{2}dz]\end{array}$$

where *Re* corresponds to the real part of the formula. *C* is an optical parameter that depends on the optical properties of the BT layer and on those of the mica substrate and the details are given in the Supplementary Note 2. The subscripts 1 and 2 define the BT and mica medium and *r*_12_ is the optical reflectivity coefficient at the interface BT/mica. *k*_1_ is the probe wavevector in the BT medium. The first term in the formula is the contribution of the interferometric effect due to the thickness change induced by the coherent acoustic phonon strain field and the second term corresponds to the photoelastic contribution, i.e. that due to the modulation of the refractive index by the coherent acoustic phonons strain field. This strain field is coupled to the internal probe light electric field given as the second term (into the brackets) in the integral term. We remind that z = 0 corresponds to the free surface of BT layer (we have neglected the contribution of the passivation oxide layer at this level). $\frac{\partial {k}_{1}}{\partial {\eta }_{{\rm{33}}}}$ is the photoelastic coefficient. We can derive the latter one according to ${k}_{{\rm{0}}}\frac{\partial {n}_{{\rm{1}}}}{\partial {\eta }_{{\rm{33}}}}={k}_{{\rm{0}}}\frac{\partial {n}_{{\rm{1}}}}{\partial {E}_{{\rm{probe}}}}\times \frac{\partial {E}_{{\rm{probe}}}}{\partial {\eta }_{{\rm{33}}}}={k}_{{\rm{0}}}\frac{\partial ({n^{\prime} }_{{\rm{1}}}+i{n^{\prime\prime} }_{{\rm{1}}})}{\partial {E}_{{\rm{probe}}}}\times \frac{\partial {E}_{{\rm{probe}}}}{\partial {\eta }_{{\rm{33}}}}$, where *k*_0_ and $\frac{\partial {E}_{{\rm{probe}}}}{\partial {\eta }_{{\rm{33}}}}$ are the optical wavevector in vacuum and the deformation potential coefficient at the probe energy *E*_probe_ and ${n}_{{\rm{1}}}={n^{\prime} }_{{\rm{1}}}+i{n^{\prime\prime} }_{{\rm{1}}}$ is the BT refractive index (${n}_{{\rm{1}}}=1.7+i4.5$ at the probe energy^32^). From the literature^32^ one can extract both the real and the imaginary part of the derivative of the refractive index at the probe energy (2.2 eV) and we found that $\frac{\partial n{^{\prime\prime} }_{{\rm{1}}}}{\partial {E}_{{\rm{probe}}}}/\frac{\partial n{^{\prime} }_{{\rm{1}}}}{\partial {E}_{{\rm{probe}}}}\sim 1.5$. The refractive index of Mica was take*n* as *n*_2_ = 1.6. The result of the calculation gives a time-dependent sinusoidal function for transient optical reflectivity signal with the corresponding frequency of the eigenmode. We focus on the thickness dependence of the magnitude of this signal. This maximum amplitude is not the absolute one, since *A* as well as $\frac{\partial {E}_{{\rm{probe}}}}{\partial {\eta }_{{\rm{33}}}}$ (deformation potential at the probe energy) are not known. But, importantly, we can discuss the thickness *L* dependence at least. Considering that the sound velocity of the BT layers does not change (see Fig. 1d), we can say that *A* is proportional to the electron-hole acoustic deformation potential (*d*_ac-eh_) and the photoexcited carriers concentration *N* only as:^45–47^

5 $${\eta }_{33}(z,t)=A\times sin[\frac{\pi z}{L}]{e}^{i{\omega }_{0}t}\propto N(L)\times {d}_{33}^{{\rm{a}}{\rm{c}}-{\rm{e}}{\rm{h}}}\times sin[\frac{\pi z}{L}]{e}^{i{\omega }_{0}t}$$

As shown in Fig. 1c, the Beer-Lambert approach appears reasonable which shows that the optical properties do  not drastically change at the probe energy. We can then estimate how *N* varies versus the layer thickness *L* and show that *N* is roughly multiplied by two in a layer of 4 nm compared to a 10 nm thick one. We have taken this into consideration in the calculation.

The calculated thickness dependence of the maximum of amplitude of the detected eigenmode is shown in Fig. 4b (red dashed curve). A comparison is given with the experimental amplitude (black dots) of the maximum of the eigenmode oscillation (see comparison of some signals in the inset of Fig. 4b). As mentioned before, we cannot compare the absolute values, but only *L* variations; as matter of fact we have normalized the curve to their maximum for discussing the tendency. It appears that the detection mechanism reproduce an enhancement of the detection of the coherent acoustic phonons as well as a decrease of the detected signal for ultrathin layers. If now we include in this calculation the thickness dependence of the deformation potential, we obtain the green curve (curve labelled as model). Some similitude in the thickness dependence appears but there is a clear shift between theory and experiments that might comes from the approximations we did. Among them, we have used the $L\frac{\partial {E}_{{\rm{g}}}}{\partial L}$ parameter of BS due to the lack of enough precise data for BT. Moreover, we considered the photoelastic coefficient as thickness independent which is very likely not true.

At this level we do not claim that we have definitive conclusion but we have to admit that the comparison between experiments and the theoretical calculation, supports a quantum size effect on the electron-hole acoustic phonon deformation potential as never reported before. As an intermediate important conclusion, this model could establish a possible partial correlation between the size dependent electron-hole acoustic phonon deformation potential and the observation of the increase of the carrier relaxation rate, in the case of predominant scattering of carriers by the acoustic phonons. As discussed above, surface trapping also play probably a role. Moreover, it is worth to underline that our model (Eq. 3) is related only to bulk electronic level and does not take into account the electron-hole acoustic phonon coupling with surface states. Recent theoretical consideration done by Giraud *et al* ^27^. have shown that the quantum size effect leads also to an enhancement of the Dirac surface electron-acoustic phonon coupling which shows that both approaches go in the same direction suggesting an enhancement of coherent acoustic phonon generation in quantum-confined BT layer. This size dependence of the electron-hole acoustic phonon deformation potential parameter needs to be confirmed with ab-initio calculations.

As a conclusion, this work reveals a clear modification of the out-of-equilibrium carriers and phonons dynamics when the BT layer is reduced to few nanometers. This time-domain investigation provides a new insight in the size-dependent physical properties of topological insulators while size-dependent electronic properties were probed in the past only at the thermodynamic equilibrium. While known for semiconductor and metallic nanostructures up to now, we report a similar drastic decrease of the electron-phonon relaxation time with an increase of the confinement. We can assign this effect to a size-dependent electron-hole phonon deformation potential parameter which need to be confirmed by ab-initio calculation. In particular, the increase of the coherent acoustic phonons signals with decreasing *L* can be explained at least partially by a enhancement of the electron-hole acoustic deformation potential parameter according to our theoretical proposition. The experimental optical measurement integrates bulk and surface electrons, but non negligible surface carriers contribution could exist since our film thickness scales with the required characteristic distance for surface Dirac fermions states to hybridize^18^, but it is difficult to give a quantitative estimate by now of separated contributions. Apart this quantum-confinement effect, one cannot exclude the contribution of the carrier surface recombination that could enhance the carrier relaxation as well. All these new physical insights show that downscaling the topological insulators properties is not straightforward and these new experimental reports have to be taken into account for potential TIs based spintronic nanodevices.

## Methods

### Sample Preparation

The growth of ultrathin BT layers was performed in the co-deposition mode. The electronic and crystallographic characterizations were carried out *in-situ* with the use of XPS (Fig. 1d) and the low energy electron diffraction (LEED) (Fig. 1b). The samples were deposited on the (110) freshly cleaved Muscovite mica substrate (Ted Pella, Inc.), in the MBE chamber with mechanical shutter to realize the step and wedge geometries and to control the thickness gradient. Deposition rate was controlled with quartz crystal micro balance, which permits to estimate the thickness with accuracy of ±0.2*nm*. Stoichiometry analysis was done by XPS analysis and details are given in the SI. Static optical transmission (T) measurements were done on the wedge sample to evidence the gradient of the thickness (Fig. 1(c)). From the thickness of 10 nm down to around 2 nm, the optical transmission increases following well the Beer-Lambert law ${I}_{T}={I}_{0}{e}^{-L/\xi }$, with $\xi$ = 10 nm at an optical wavelength of 582 nm^32^, and I_0_ is the transmitted intensity with only the mica substrate. In the very thin regions of the wedge sample (less than 4 nm) some deviations are observed probably due to the electronic properties changes as revealed by XPS (Fig. 1d).

### Time-resolved optical measurements

The pump-probe technique used here is based on a 80 MHz repetition rate Ti:sapphire femtosecond laser (120 fs). The pump wavelength is fixed at the harmonic of the Ti:sa laser (830 nm, 1.495 eV) while the probe beam is introduced in a synchronously pumped OPO to tune the wavelength to 582 nm (2.13 eV). The experiments were conducted with the front-front configuration with incident pump and probe beams perpendicular to the surface as shown in Fig. 1 but also in transmission geometry (not shown) since mica substrate is transparent to both pump and probe. Pump-probe experiments were conducted with the wedge sample (passivated) and the step sample 1 (passivated). The pump (1.495 eV) and probe (2.13 eV) absorption lengths are ~10.1 nm and ~9.8 nm respectively^32^. This very small penetration depth is due to the specific electronic band structure in this energy range where interband transitions exist^32^. The maximum fluence was 100 *μ*J.cm^−2^ which corresponds to a photoexcited carriers concentration of around 10^16^ cm^−3^. Furthermore, in the experiments, the pump and probe are focused with a microscope objective providing a typical spot radius of ≈5 micrometers. With this spot diameter and sample length (along the gradient) of around 1.5 cm (very smooth gradient for the wedge sample) and a width of 1 cm, we were able to investigate many positions along and perpendicular to the gradient which permitted to get a very good statistic of transient reflectivity signals, crucial to reveal subtle properties changes.

### Data availability

The authors declare that the data supporting the findings of this study are available within the article and its Supplementary Information.

## Electronic supplementary material

Supplementary Information

## References

[1] Moore JE (2010). The birth of topological insulators. Nature.

[2] Hazan MZ, Kane CL (2010). *Colloquium*: Topological Insulators. Rev Mod Physics.

[3] Kondou K (2016). Fermi-level-dependent charge-to-spin current conversion by Dirac surface states of topological insulators. Nature Phys..

[4] Hajlaoui M (2014). Tuning a Schottky barrier in a photoexcited topological insulator with transient Dirac cone electron-hole asymmetry. Nature Comm..

[5] Mittleman DM (1994). Quantum size dependence of femtosecond electronic dephasing and vibrational dynamics in CdSe nanocrystals. Phys. Rev. B.

[6] Allan G, Delerue C (2004). Confinement effects in PbSe quantum wells and nanocrystals. Phys. Rev. B.

[7] Stella A (1996). Size effects in the ultrafast electronic dynamics of metallic tin nanoparticles. Phys. Rev. B.

[8] Arbouet A (2003). Electron-Phonon Scattering in Metal Clusters. Phys. Rev. Lett..

[9] Kirchmann PS (2010). Quasiparticle lifetimes in metallic quantum-well nanostructures. Nature Phys..

[10] Zhang Y (2010). Crossover of the three-dimensional topological insulator Bi2Se3to the two-dimensional limit Nature Phys..

[11] Vidal F (2013). Photon energy dependence of circular dichroism in angle-resolved photoemission spectroscopy of Bi2Se3 Dirac states*Phys*. Rev. B.

[12] Li Y-Y (2010). Intrinsic Topological Insulator Bi 2 Te 3 Thin Films on Si and Their Thickness Limit. Adv. Mater..

[13] Lu H-Z, Shan W-Y, Yao W, Niu Q, Shen S-Q (2010). Massive Dirac fermions and spin physics in an ultrathin film of topological insulator. Phys. Rev. B.

[14] Linder J, Yokoyama T, Sudbo A (2009). Anomalous finite size effects on surface states in the topological insulator Bi_2_Se_3_. Phys. Rev. B.

[15] Zhou B (2008). Finite Size Effects on Helical Edge States in a Quantum Spin-Hall System. Phys. Rev. Lett..

[16] Liu C-X (2010). Oscillatory crossover from two-dimensional to three-dimensional topological insulators. Phys. Rev.B.

[17] Kim YS (2011). Thickness-dependent bulk properties and weak antilocalization effect in topological insulator Bi_2_Se_3_. Phys. Rev. B.

[18] Kim D (2012). Surface conduction of topological Dirac electrons in bulk insulating Bi_2_Se_3_. Nat. Phys..

[19] Glinka YD (2013). Ultrafast carrier dynamics in thin-films of the topological insulator Bi_2_Se_3_. Appl. Phys. Lett..

[20] Kim S. *et al*. Resonance effects in thickness-dependent ultrafast carrier and phonon dynamics of topological insulator Bi_2_Se_3_ Nanotech. **27** 045705 (2016) 10.1088/0957-4484/27/4/045705 26655693

[21] Sobota JA (2012). Ultrafast Optical Excitation of a Persistent Surface-State Population in the Topological Insulator Bi_2_Se_3_. Phys. Rev. Lett..

[22] Hsieh D (2011). Selective Probing of Photoinduced Charge and Spin Dynamics in the Bulk and Surface of a Topological Insulator. Phys. Rev. Lett..

[23] Wang YH (2012). Measurement of Intrinsic Dirac Fermion Cooling on the Surface of the Topological Insulator Bi_2_Se_3_ Using Time-Resolved and Angle-Resolved Photoemission Spectroscopy. Phys. Rev. Lett..

[24] Hajlaoui M (2012). Ultrafast Surface Carrier Dynamics in the Topological Insulator Bi_2_Te_3_. Nano Lett..

[25] Onishi Y (2015). Ultrafast carrier relaxation through Auger recombination in the topological insulator Bi_1,5_Sb_0.5_Te_1.7_Se_1.3_. Phys. Rev. B.

[26] Golias E, Sanchez-Barriga J (2016). Observation of antiphase coherent phonons in the warped Dirac cone of Bi_2_Te_3_. Phys. Rev. B.

[27] Giraud S, Kundu A, Egger R (2012). Electron-phonon scattering in topological insulator thin films. Phys. Rev. B.

[28] Rapacz R, Balin K, Nowak A, Szade J (2014). Spectroscopic characterization of high-purity polycrystalline BiTe films grown by thermal evaporation. J. of Cryst. Growth.

[29] Seah MP, Specer SJ (2002). Ultrathin SiO_2_ on Si II. Issues in quantification of the oxide thickness. Surf. Interf. Anal..

[30] Hatch RC (2011). Stability of the Bi_1_Se_3_(111) topological state: Electron-phonon and electron-defect scattering. Phys. Rev. B.

[31] Park BC (2015). Terahertz single conductance quantum and topological phase transitions in topological insulator Bi_2_Se_3_ ultrathin films. Nat. Comm..

[32] Greenaway DL, Harbere G (1965). Band structure of bismuth telluride and their alloys. J. Phys. Chem. Solids.

[33] Weis M (2015). Ultrafast Light-Induced Coherent Optical and Acoustic Phonons in few Quintuple Layers of Topological Insulators Bi_2_Te. Phys Rev. B.

[34] Flock J, Dekorsy T, Misochko OV (2014). Coherent lattice dynamics of the topological insulator Bi2Te3 probed by ultrafast spectroscopy. App. Phys. Lett..

[35] Kumar N (2011). Spatially resolved femtosecond pump-probe study of topological insulator Bi_2_Se_3_. Phys. Rev. B.

[36] Wang Y, Guo L, Xu X, Pierce J, Venkatasubramanian R (2013). Origin of coherent phonons in Bi_2_Te_3_ excited by ultrafast laser pulses. Phys. Rev. B.

[37] Eddrief M, Atkinson P, Etgens V, Jusserand B (2014). Low-temperature Raman fingerprints for few-quintuple layer topological insulator Bi_2_Se_3_ films epitaxied on GaAs. Nanotech..

[38] Glinka YD, Babakiray S, Lederman D (2015). Plasmon-enhanced electron-phonon coupling in Dirac surface states of the thin-film topological insulator Bi_2_Se_3_. J. Appl. Phys..

[39] Shahil KM, Hossain MZ, Teweldebrhan D, Balandin AA (2010). Crystal symmetry breaking in few-quintuple Bi_2_Te_3_ films: Applications in nanometrology of topological insulators. Appl. Phys. Lett..

[40] Giret Y, Gellé A, Arnaud B (2011). Entropy Driven Atomic Motion in Laser-Excited Bismuth. Phys. Rev. Lett..

[41] Johnson SL (2008). Nanoscale Depth-Resolved Coherent Femtosecond Motion in Laser-Excited Bismuth. Phys. Rev. Lett..

[42] J. M. Ziman, *Principle of Theory of Solids*, Cambridge University Press, 1964.

[43] Huang B-L, Kaviany M (2008). Ab initio and molecular dynamics predictions for electron and phonon transport in bismuth telluride. Phys. Rev. B.

[44] Thomsen C, Grahn HT, Maris HJ, Tauc J (1986). Surface generation and detection of phonons by picosecond light pulses. Phys. Rev. B.

[45] Yu, P. & Cardona, M. Fundamentals of Semiconductors, Springer Verlag Heidelberg (1996).

[46] Gusev V. and Karabutov A. *Laser Optoacoustics*, AIP, New York (1993).

[47] Ruello P, Gusev V (2015). Physical mechanisms of coherent acoustic phonons generation by ultrafast laser action. Ultrasonics.

[48] Wright OB (1992). Thickness and sound velocity measurement in thin transparent films with laser picosecond acoustics. J. Appl. Phys..

[49] Gusev V (1996). Acustica Acta Acustica.

[50] Matsuda O, Wright OB (2003). Laser picosecond acoustics with oblique probe light incidence. Rev. Sci. Instrum..

