# Supplementary material for: Quantum size effect on charges and phonons ultrafast dynamics in atomically controlled nanolayers of topological insulators Bi2Te3
Source: Sci Rep. 2017 Oct 23;7:13782. doi: 10.1038/s41598-017-12920-4 (PMC5653873; doi:10.1038/s41598-017-12920-4)
Supplement: Supplementary file 1 — Supplementary Information [file 41598_2017_12920_MOESM1_ESM.pdf]

Quantum size effect on charges and phonons ultrafast dynamics in atomically controlled nanolayers of topological insulators Bi_2Te_3.

M. Weis[‡,†], B. Wilk[‡,†], G. Vaudel[†], K. Balin[‡], R. Rapacz[‡], A. Bulou[†], B. Arnaud [†], J. Szade[‡*], P. Ruello[† †]
[‡] *A. Chełkowski Institute of Physics and Silesian Center for Education and Interdisciplinary Research, 75 Pułku Piechoty 1A, 41-500 Chorzów, University of Silesia, Poland.*
[†] *Institut des Molécules et Matériaux du Mans, UMR CNRS 6283, Université du Maine, 72085 Le Mans, France.*

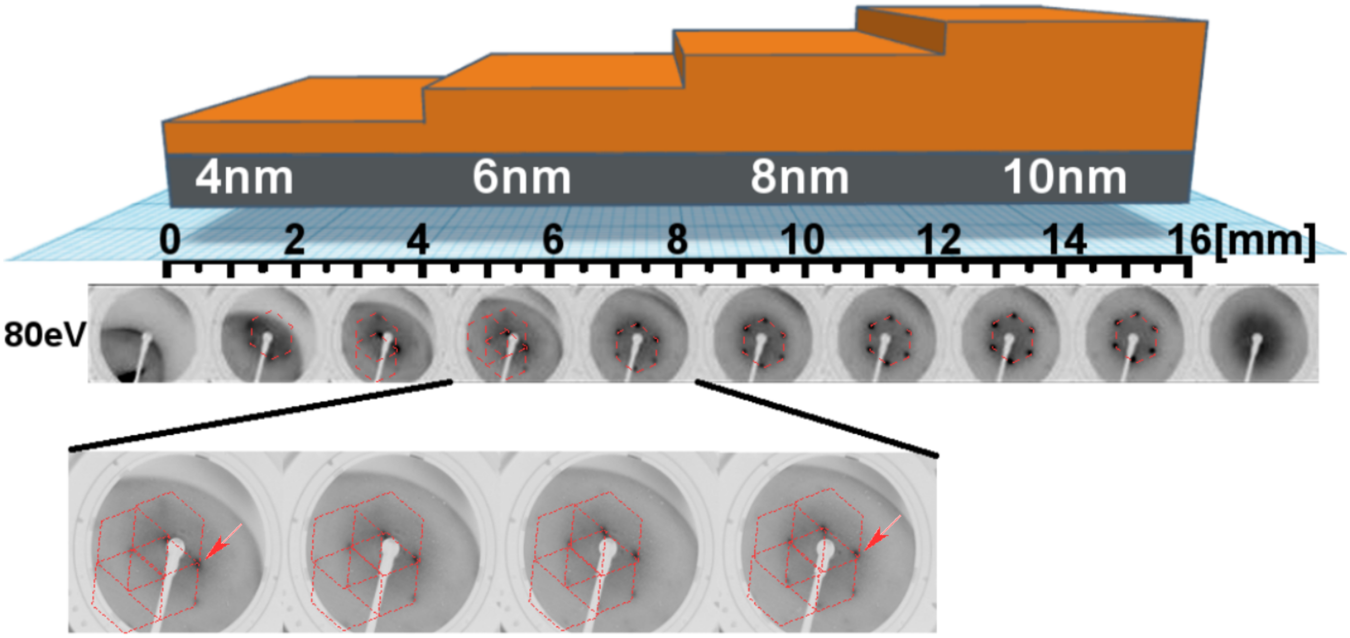

FIG. 1: Low Energy Electron Diffraction (LEED) measurements were conducted every 1mm along the sample with energy of 80eV (step sample 1).

* Electronic address: jacek.szade@us.edu.pl
† Electronic address: pascal.ruello@univ-lemans.fr

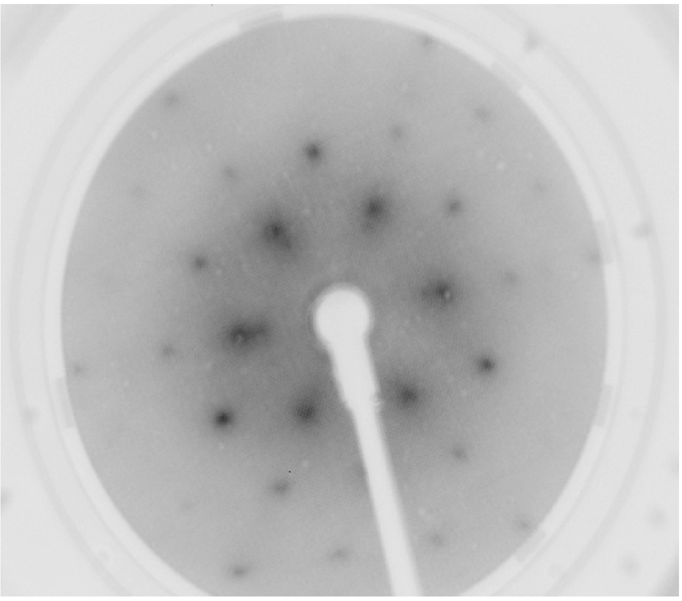

FIG. 2: LEED of 5nm thickness Bi_2Te_3 sample at an energy of 115eV.

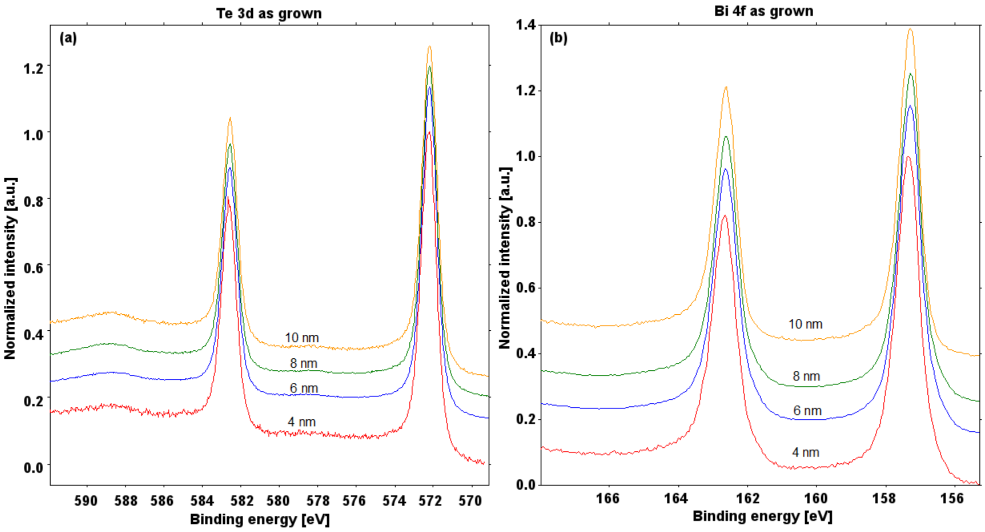

FIG. 3: X-ray Photoemission Spectroscopy of Te 3d (a) and Bi 4f (b) doublets for the as-grown films in the step sample 1.

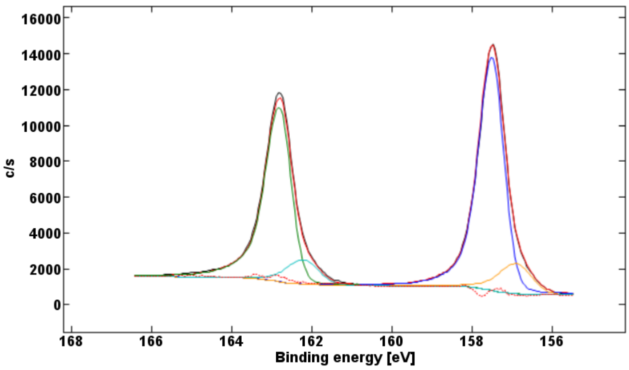

FIG. 4: Fit of the Bi4f doublet for the 6 nm thick film showing the contributions from the Bi_2Te_3 (high intensity doublet) and Bi (low intensity doublet). These data concern the step sample 1.

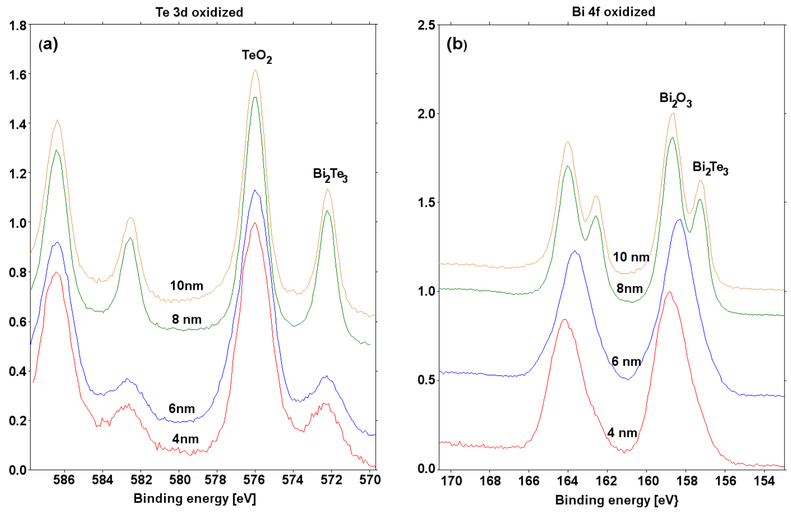

FIG. 5: XPS doublets of Te 3d (a) and Bi 4f (b) after oxidation of the film.

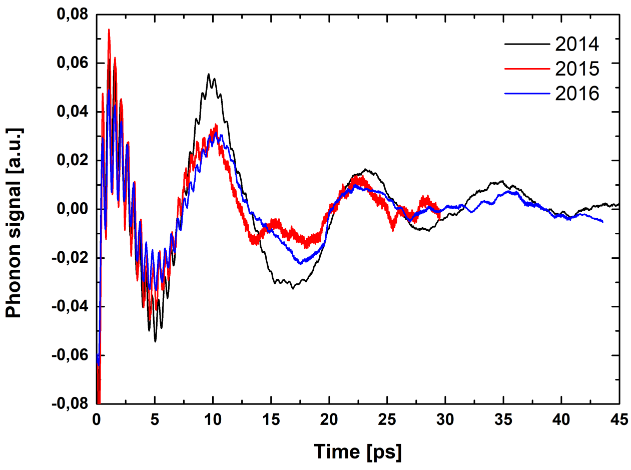

FIG. 6: Evolution in time of the optical and acoustic phonon signal

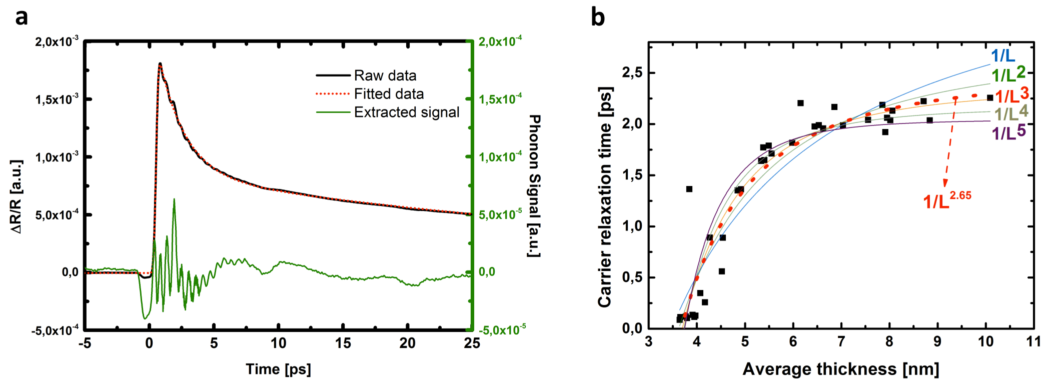

FIG. 7: (a) Measured signal was fitted with function Eq. 1. Extracted this way signal is representing mainly the phonon signal from investigated region. (b) Relaxation time fitted with different powers of law. The best result of fitting is closest to L^3.

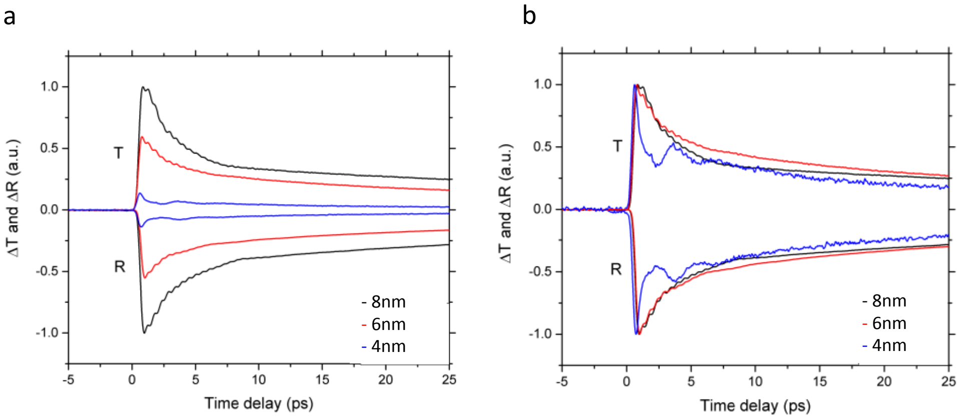

FIG. 8: (a) Comparison between transient optical transmission and reflectivity signals obtained for step sample 1. (b) Normalized signals to the maximum of the transient change (i.e. to the electronic peak) revealing a clear change of electron and phonon dynamic for ultrathin layers.

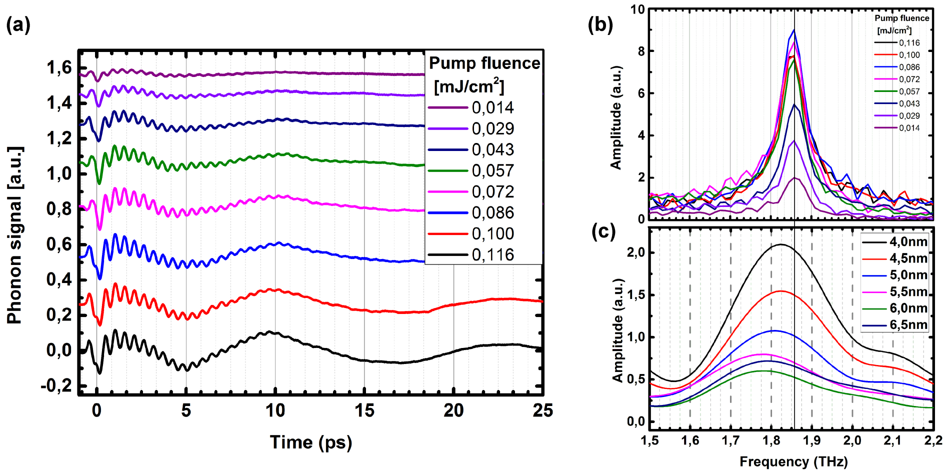

FIG. 9: (a) Oscillatory components $\Delta R/R_{phonon}$ of the transient optical reflectivity signals obtained for variable pump fluence for a film of 15 nm of thickness. (b) Fast Fourier transform of the signals (a). No detectable softening of the A1g mode appears contrary to the confinement effect (c) where A1g mode frequency in ultra thin film clearly softens.

I. SUPPLEMENTARY NOTE 1 : GROWTH AND SURFACE CHARACTERIZATION

A. LEED measurements

Low energy electron diffraction experiments were conducted on the step layer sample 1 (in-situ, i.e. before oxidation) in order to confirm proper growth of the crystalline layer (Fig.1). Measurements confirm the proper BT symmetry whatever the thickness (4, 6, 8 or 10 nm). In the region of 4nm image is distorted because of the bending of the mica substrate. It is worth to mention that in the region (thickness typically smaller than <6 nm) corresponding to the observed drastic change of the electron relaxation and to the modification of the phonons dynamic, the obtention of the proper symmetry of Bi_2Te_3 has been checked. LEED measurements were conducted as well in two other energies 85eV and 100eV

To further assure the proper crystallization additional film with a thickness of 5 nm was grown on a mica substrate in exactly the same conditions as other samples. The corresponding LEED image is shown in Fig. 2 confirming the right symmetry is obtained.

B. XPS characterization

Photoelectron spectroscopy studies were performed with the use of the monochromatized AlKα radiation in the PHI 5700 spectrometer from Physical Electronics. The as grown film were transferred in UHV from the MBE to the analysis chamber within minutes. The films studied in-situ showed no oxidation nor carbon contamination. The analysis spot had the diameter of 0.8 mm. The analysis of the spectra was done with the use of MULTIPAK program from Physical Electronics. The lines were fitted by Gaussian-Lorentz or asymmetric lines. The same program was used to calculate the atomic concentration.
The Te 3d and Bi 4f doublets and valence band were used for analysis the step sample 1. The core levels of Te 3d doublet (Fig. 3(a)) and those of the Bi4f levels (Fig. 3(b)) confirm that whatever the thickness, the electronic structure of BT is obtained. However, a small contribution from metallic Bi could be detected after fitting of the spectra for the films with thickness up to 8 nm. Fig. 4 shows the example of the results of fitting for the 6 nm film. This fact correlates with the obtained atomic concentration which is rich in Bi especially for the thickness of 4 and 6 nm (Table 1).

Table 1. Atomic concentration for the as grown films

Thickness	Tellurium	Bismuth
4 nm	49,5	50,5
6 nm	52,4	47,6
8 nm	52,1	47,9
10 nm	53,2	46,8

One of the possible reasons of the Te deficiency changing with the thickness of the films is related with the desorption of Te at the initial stage of growth. Formation of additional Bi layers between QLs is a consequence of that process. The superstructure Bi-BT conserves the stable structure within the QLs as shown in a previous work [1]. The Te desorption may be related to latent heat of the compound formation which causes overheating of the deposited film. The effect will be most pronounced at the thinnest films because the heat transfer to the substrate is limited and the mass of the deposited BT is very low. The interface BT-mica is of the van der Waals character limiting the heat transfer to the substrate.

As mentioned in the main text, the pump-probe investigations were conducted with oxidized thin BT films (ex-situ investigation). As discussed below, we have checked that the oxidation passivates the film without disturbing the electronic structure of the BT layer. In order to estimate the effect of surface oxidation we performed the XPS analysis few months after the growth. The analysis has shown that even for the thinnest part of the sample we could indeed observe in the photoemission core level spectra the contribution from the unaffected BT compound with characteristic position of the Te and Bi lines (Fig. 5). The remains of the Bi interlayers could also be detected for the films 4 and 6 nm thick. The oxides formed on the surface are mostly Bi_2O_3 and TeO_2. For the thinnest films a small contribution with higher binding energy than for TeO_2 was observed indicating to TeO_3.
To estimate the thickness of the oxide layer we used the formula taking into account photoemission intensity damping [1]:

$$\frac{I_C}{I_F} = \frac{I_{C_0}\left[1 - exp\left(\frac{-d}{\lambda_C cos\Theta}\right)\right]}{I_{F_0} exp\left(\frac{-d}{\lambda_F cos\Theta}\right)}$$

The intensity ratio from the oxide cap layer (I_C) and the main film (I_F) can be expressed with the use of the thickness of the cap layer (d) and inelastic mean free path within the basic film (λ_F) and the cap layer(λ_C). The analysis angle was 45°.
The photoelectron mean free path λ can be estimated basing on the TPPM2M formula using the Quases program of S. Tougaard [2–4]. It is about 1.75 nm for the TeO_2 and 2.25 nm for Bi_2O_3. We calculated the relative intensity of the photoemission components coming from oxides and BT. It changes with the thickness, while for thin films of 4 and 6 nm the intensity ratio oxide to BT is between 3 and 6, for the thicker films it is around 1.8.
When assuming uniform cap layer of mixed Bi and Te oxides one can calculate the thickness of the oxide film. Mean free path of about 2 nm was taken to the formula:

$$d = \lambda cos\Theta ln\left(\frac{I_c}{I_F} + 1\right)$$

where we applied assumption $I * C_0 /\ I_{F_0}$=1. The calculated thickness of the oxide layer varies from about 2.2 nm for thin films to about 1.4 nm for the films with thicknesses of 8 and 10 nm.

Table 2. Atomic concentration for the oxidized films

Thickness	Tellurium	Bismuth	C	O
2 nm	8.3	9.3	45.0	37.4
4 nm	10.5	11.0	41.1	37.4
6 nm	12.2	13.0	40.9	33.9
8 nm	9.5	10.2	47.7	32.8

C. Evolution in time of the light-induced phonons signal

In order to confirm the XPS investigation, we have studied how the transient phonon signal evolves in time. The details of the extraction of the signal is given below in the Supplemnetary Note II of the Supplementary Information. We show in Supplementary Figure 6 how the optical and acoustic phonon signals change in time. These signals are obtained on a film with a nominal thickness of 15 nm that was grown in the same conditions as the one used for the step and the wedge samples of the paper. This sample was grown in 2014 and then it offers the opportunity to see how it evolves in time. In order to reveal better the phonon signal we show the derivative of the signal only. We see clearly the optical phonon signal with no variation of the A1g frequency. Furthermore, we can note that a slight degradation is observed on the acoustic phonon signal (long period) but the frequency hardly changes. The slight evolution is likely be due to a part of the manipulation of the sample on a brittle mica substrate, but also due to natural aging. This comparison is also done with three measurements that were not done exactly on the same position of the surface which might also slightly contribute to the difference. The most important thing is that the acoustic phonon oscillation period does not vary significantly which indicates that the thickness of the resonator (BT layer) neither significantly changes in time. As a consequence, this is a very good indication that the oxide layer is stable, else the resonator period would have been altered more seriously.

D. AFM characterization

Surprisingly the AFM studies of the oxidized sample showed higher roughness for the thicker films. It may be related to the Stransky-Krastanov way of BT growth leading to the increased differences between the columns height. The quality of the surface seems to be better for thin films which, as a consequence, could have a the cap oxide layer with a smaller thickness. Nevertheless, the oxides cap layer (between 1.4 and 2.5 nm) clearly protects the film against further oxidation. It does not influence or hardly influences the optical response of the main film composed of Bi_2Te_3 with a possible interlayers of Bi.

Table 3. AFM roughness measurements on step sample after oxidation

Thickness	Roughness
8 nm	2.25 nm
6 nm	0.57 nm
4 nm	0.31 nm
2 nm	0.68 nm

The obtained thickness of the oxides film on the BT films means that the real BT layer thickness of the compound (ex-situ experiments) is smaller than that from the the as-grown films by about 2 nm.

II. SUPPLEMENTARY NOTE 2 : ULTRAFAST DYNAMICS

A. Electronic decay fitting procedure

As earlier proposed in the literature [7], the transient electronic contribution $\Delta R/R_{elec.}$ in the optical reflectivity signals can be numerically adjusted with a function that takes into account the time dependent integrated response of the pump-probe cross correlation (erf function) as well as the decaying signal. For the latter one, we need two use two characteristic time, τ_1 for the fast dynamic (ps) and τ_2 for the slower one (thermal effect or slow electronic relaxation).

$$\frac{\Delta R\left(t\right)}{R}_{elec.} = \left[1 + erf\left(\frac{t}{\sigma} - \frac{\sigma\left[\tau_1 + \tau_2\right]}{\tau_1\tau_2}\right)\right]\left[A_1 exp\left(-\frac{t}{\tau_1}\right) + A_2 exp\left(-\frac{t}{\tau_2}\right)\right] \tag{1}$$

with σ the resolution of measurement, τ_1 the hot carrier relaxation, τ_2 the thermal relaxation, A_1 the hot carriers contribution and A_2 the thermal contribution. A typical numerical adjustment is shown by the red curve in Fig. 1(a). Once the electronic contribution adjusted, it is possible to extract only the phonon contribution $\Delta R/R)_{phonon.}$ arising from the optical and acoustic phonons (green curve in Fig. 1(a)).

Regarding the thickness dependence of the relaxation time, we have adjusted the τ_1 time with a law $1/L^{\alpha}$ as shown in Fig. 7(c). Relaxation time of carrier is given by quantum well length as follows:

$$\tau_1\left(L\right) = \tau_0^B - \frac{A}{L^{\alpha}} \tag{2}$$

τ_0^B- relaxation through bulk structure
$A-$ scaling factor
$L-$ length of quantum well

B. Transient optical transmission versus transient optical reflectivity:

In order to show that the size dependence of the electronic and phonon signal is intrinsic to the BT film, we have carried out time-resolved optical transmission as well. This was possible since the substrate (Mica) is transparent and the BT layer is thin enough. We remind here that the optical skin depth at the probe energy of 2.2eV is around 10 nm, so enough light energy goes is transmitted. In Supplementary Figure 8, we compared the raw signal (a) and those normalized to the maximum of the transmission change (i.e. to the so-called electronic peak).

C. Pump power dependence and A1g mode softening :

A pump power dependence study was realized on a film of thickness 15 nm. We have observed that the thicker film has a damage threshold larger than for the thin layers. This is likely due a better evacuation of heat accumulated with high repetition lasers. As a consequence, it was more easy to span a large pump power range for thick film. The pump dependence of the phonons contribution to the transient optical reflectivity signal is shown in Fig. 9(a) for an

energy range of 0.01- 0.1 mJ/cm^2. The A1g phonon signal amplitude increases but there is no detectable shift of the A1g mode. This is consistent with the absence of an electronic temperature effect as known for Bismuth for example [5, 6]. In order to compare the thick sample (15 nm) with the ultrathin ones, we have installed in the bottom of Fig. 9(b) the thickness dependence of the A1g frequency. This comparison clearly evidence the mode softening in presence of confinement.

D. Transient optical reflectivity calculation

The calculation of the coherent acoustic phonon contribution to the transient optical reflectivity has been performed following the standard model where the optical reflectivity magnitude for normal incidence pump-probe experiment is $\Delta R/2R = Re(\Delta r\ /r)$ with [8–10]:

$$\begin{aligned}\Delta R/2R &= Re(C(2ir_{12}k_1(u(0)-u(L)) \\ &- i\frac{\partial k_1}{\partial \eta_{33}}\int_0^L \eta_{33}(z,t)[r_{12}e^{-ik_1(L-z)}+e^{ik_1(L-z)}]^2 dz \\ &- i(1-r_{12}^2)\frac{\partial k_2}{\partial \eta_{33}}\int_L^\infty \eta_{33}(z,t)e^{i2k_2z}dz]))\end{aligned} \tag{3}$$

with

$$C = \frac{(1-r_{01}^2)}{(r_{01}e^{ik_1H}+r_{12}e^{-ik_1H})}\times\frac{1}{(e^{ik_1H}+r_{01}r_{12}e^{-ik_1H})} \tag{4}$$

Re means the real part, r is the complex optical reflectivity coefficient of the non-perturbated system, k_i is the probe wavevector in medium i (0, 1 and 2 are respectively air, BT layer and the Mica substrate), $\eta_{33}(z,t)$ is the acoustic strain, L is the thickness of the BT layer and *u(z)* is the normal mechanical displacement of the transparent layer at a position z. The notation r_ij is used for the optical reflection coefficient at the interface between medium i/j for oblique incident probe beam whose polarization is perpendicular to the incidence plane :

$$r_{ij} = \frac{n_i - n_j}{n_i + n_j} \tag{5}$$

The first term of Eq. (3) corresponds to an interferometric contribution due to the change of the transparent layer thickness (*u(0)-u(H)*) induced by the relative interfaces displacement driven by the acoustic field. In our calculation (see main manuscript) only the first acoustic eigenmode was taken into account. A straigthforward spatial integration from 0 to L of the strain $\eta_{33}(z,t) = Asin[\frac{\pi z}{L}]e^{i\omega t}$ gives $2ir_{12}k_1(u(0)-u(L))e^{i\omega t} = 2ir_{12}k_1\int_0^L \eta_{33}(z,t)dz = -4i\times r_{12}k_1\frac{L}{\pi}e^{i\omega t}$. The first integral term is the photoelastic contribution due to the scattering of the probe beam electric field by the acoustic strain $\eta_{33}(z,t)$ within the transparent layer (photoelastic contribution). The second integral term comes from the photoelastic contribution in the Mica substrate. In our calculation, since no evidence of coherent acoustic phonons signals from the mica substrate was evidenced, we have neglected this photoelastic contribution. Moreover, the BT is attached to the mica substrate by Van der Waals bonds which must limit the transmission of coherent acoustic phonon inside the substrate. In our calculation, we also did not take into account the contribution of the vibration of the thin passivation layer that covers the BT layer. This is supported by the fact that transparent materials such as oxide and mica have smaller photoelastic coefficient than opaque materials such as BT and this in particular in the case of detection close to an interband transition of the BT layer where photoelastic coefficient are usually enhance [11]. Considering the photoelastic coefficient, one can derive it as $k_0\frac{\partial n_1}{\partial \eta_{33}} = k_0\frac{\partial n_1}{\partial E_\mathrm{probe}}\times\frac{\partial E_\mathrm{probe}}{\partial \eta_{33}}$, where k_0 and $\frac{\partial E_\mathrm{probe}}{\partial \eta_{33}}$ are the optical wavevector in vacuum and the deformation potential coefficient at the probe energy E_probe. From the literature [12] one can extract both the real and the imaginary part of the derivative of the refractive index at the probe energy (2.2 eV) and we found that $\frac{\partial n_1''}{\partial \eta}/\frac{\partial n_1'}{\partial \eta}\sim 1.5$ ($n_1 = n_1' + in_1'' = 1.7 + i4.5$ at the probe energy [12]). The result of the calculation gives a time-dependent sinusoidal function for transient optical reflectivity signal and we focus on the magnitude of this signal. This later one is not the absolute one, since A as well

as $\frac{\partial E_{\text{probe}}}{\partial \eta_{33}}$ (deformation potential at the probe energy) are not known in absolute. But, importantly, we can discuss the thickness L dependence.

---

[1] Cava R. J., Huiwen J., Fuccillo M. K., Gibson Q. D., Hor Y.S., *J. Mater. Chem. C* **1**, 3176 (2013).
[2] Seah M.P., Specer S.J., *Surf. Interf. Anal.* **33**, 631 (2002).
[3] Tanuma S., Powell C. J., Penn D. R., *Surf. Interf. Anal.* **21**, 165 (1994).
[4] QUASES program by S. Tougaard (http://www.quases.com/home/)
[5] Giret Y., Gellé A., Arnaud B., *Phys. Rev. Lett.* **106**, 155503 (2011).
[6] Johnson S. L., *et al. Phys. Rev. Lett.* **100**, 155501 (2008).
[7] Golias E., Sanchez-Barriga J., *Phys. Rev. B* **94**, 161113(R) (2016).
[8] Wright O. B.,*J. Appl. Phys.* **71**, 1617 (1992).
[9] Gusev V., *Acustica Acta Acustica* **82**, S37 (1996).
[10] Matsuda O., Wright O. B. *Rev. Sci. Instrum.* **74**, 895 (2003).
[11] Babilotte P., Ruello P., Mounier D., Pezeril T., Vaudel G., Edely M., Breteau J-M., Gusev V, Blary K., *Phys. Rev. B.* **81**, 245207 (2010).
[12] Greenaway D. L., Harbere G., *J. Phys. Chem. Solids* **26**, 1585-1604 (1965).
